# Need for Early Recognition of Amyloidosis in Cases of Unexplained Heart Failure: A Case Report

**DOI:** 10.7759/cureus.40658

**Published:** 2023-06-19

**Authors:** Sneha Kalluri, Jamil Abbasi

**Affiliations:** 1 Internal Medicine, Baylor Scott & White All-Saints Medical Center, Fort Worth, USA; 2 Critical Care, Baylor Scott & White All-Saints Medical Center, Fort Worth, USA

**Keywords:** intensive care unit, immunoglobulin, autoimmune disease, plasma cell dyscrasias, critical care, heart failure, light chain amyloidosis, liver dysfunction, cardiogenic shock, amyloidosis (al)

## Abstract

Amyloidosis is a plasma cell dyscrasia that leads to the excessive production and deposition of mutant protein fragments in various organs. Cardiac amyloidosis is often implicated in two main subtypes: transthyretin (ATTR) and light chain (AL). While both subtypes increase the risk of restrictive cardiomyopathy, cardiogenic shock, and arrhythmias, poorer outcomes are seen in those with cardiac infiltration secondary to AL amyloidosis. Prognosis depends on the timing of diagnosis and the extent of the disease burden prior to recognition and treatment. The following case report describes a young patient who was admitted to the intensive care unit (ICU) for concerns of decompensated heart failure of unknown etiology, later determined to be due to amyloidosis. We describe her clinical course prior to and during hospital admission, along with the proposed physiologic factors that may have contributed to her poor outcome.

## Introduction

Amyloidosis refers to the abnormal deposition of protein fibrils in either a localized or systemic fashion. Localized amyloidosis typically affects the site of misfolded protein production, such as the bladder, ureter, and brain [1]. On the other hand, systemic variation is often associated with abnormal protein aggregation among several organs. Light chain (AL) amyloidosis is a systemic subtype that is caused by an immunoglobulin mutation leading to aberrant plasma cell clones [2]. These clones cause insoluble light chain fibril deposition, most notably in the liver, kidneys, and heart. While the median age at diagnosis is 64 years, the timeline of symptom presentation can vary [3]. For this reason, diagnosis is often delayed as initial symptoms are non-specific [4]. The presentation can often mimic symptoms of heart failure, such as dependent edema, shortness of breath, or abdominal distension. Up to 70% of AL cases have cardiac involvement [5], which is also found to be the primary factor contributing to increased mortality in such patients [6]. When an infiltrative process is suspected, transthoracic echocardiogram and cardiac magnetic resonance imaging (MRI) findings may be suggestive of cardiac involvement. Laboratory tests such as serum protein electrophoresis with immunofixation, urine protein electrophoresis with immunofixation, and serum-free light chain assays should be performed. These studies may help to suggest the diagnosis, but confirmation must be obtained through a biopsy of an affected organ [7]. The gold standard of testing is polarizing microscopy, which visualizes "apple-green birefringence" of tissue and/or bone marrow biopsy via Congo red stain [8]. In the following report, we present a young patient with decompensated heart failure secondary to newly discovered amyloidosis. We discuss the extent of her disease at the time of discovery and the modalities utilized for management.

## Case presentation

A 46-year-old woman was transferred to our intensive care unit (ICU) for a higher level of care. About 10 months prior to her arrival, she had presented to her primary care provider with abdominal swelling and bilateral lower extremity edema. Her only known past medical history at that time was notable for hypothyroidism and gastric bypass surgery. Lab work revealed elevated liver function tests (Table 1), which were further investigated by an abdominal ultrasound showing mild hepatomegaly with cirrhosis-appearing morphology.

**Table 1:** Laboratory values at the time of initial presentation and time of ICU admission

Laboratory value	Reference range and units	Time of ICU transfer	10 months ago (at primary care provider)
Hemoglobin	12-16 g/dL	11.6	11
Platelet count	140-440 K/uL	162	216
Albumin	3.4-5.0 g/dL	3.6	4.4
Albumin/globulin ratio	1.1-2.2	1.2	1.6
Aspartate aminotransferase (AST)	15-37 U/L	119	73
Alanine aminotransferase (ALT)	13-56 U/L	63	40
Alkaline phosphatase (ALP)	45-117 U/L	592	417
Bilirubin, total	0.2-1.0 mg/dL	8.9	0.8
Creatinine	0.55-1.02 mg/dL	0.68	0.51
Brain natriuretic peptide (BNP)	5-100 pg/mL	1,946	(not drawn)

She was started on oral furosemide and spironolactone and referred to a hepatologist for further workup of suspected hepatic disease. Viral hepatitis antigens for types A, B, and C were negative. Autoimmune testing included anti-mitochondrial antibodies, anti-nuclear antibodies, alpha-1-antitrypsin, anti-smooth muscle antibodies, and ceruloplasmin, all of which were also negative. She was advised to obtain a transjugular liver biopsy, but this was postponed due to a lack of coverage by her health insurance. She continued to manage her symptoms via diuresis for six months until she was eventually hospitalized at an outside facility for dyspnea. Though she was not hypoxic, she appeared to be hypervolemic given her lower extremity pitting edema and chest x-ray findings of central congestion. She underwent an echocardiogram, showing a reduced ejection fraction of 45% and no additional remarkable findings. Despite being discharged on increased doses of her home diuretics, she was readmitted just four months later for volume overload and hypotension. Her symptoms were suspected to be complications of liver disease; therefore, she was transferred to our facility to undergo biopsies by our interventional radiology department. On arrival, the patient was afebrile, breathing comfortably on room air in normal sinus rhythm but hypotensive with an initial mean arterial pressure of 61 mmHg (normal: 70-100 mmHg). She was placed in the intensive care unit (ICU) for a norepinephrine infusion. Her notable laboratory values on arrival showed a worsening of her liver function tests along with her total bilirubin. She was noted to have an elevated brain natriuretic peptide level, suggesting an exacerbation of her heart failure. Her kidney function, albumin, and complete blood count had remained stable from prior evaluations (Table 1).

**Figure 1:**
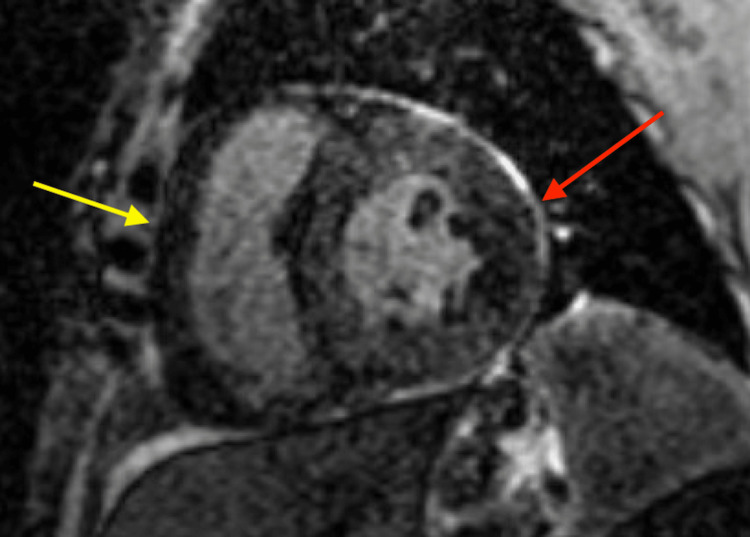
Cardiac magnetic resonance image with delayed gadolinium enhancement, displaying a thickened right ventricular myocardium (yellow arrow) and transmural thickening of the left ventricular myocardium (red arrow)

The patient underwent a liver and bone marrow biopsy. As results were pending, additional laboratory tests were performed. Serum protein electrophoresis with immunofixation was normal; however, urine protein electrophoresis revealed elevated albumin at 1,030 mg/dL (normal: 7-14 mg/dL) with a monoclonal spike (M spike) at 8.3 mg/dL (normal: none). Unfortunately, urine immunofixation was unable to be performed for the evaluation of monoclonality. Her serum kappa light chains were elevated at 30.9 mg/dL (normal: 3.3-19.4 mg/dL) with elevated lambda serum light chains at 540.4 mg/dL (normal: 5.7-26.3 mg/dL), resulting in a notably depressed kappa/lambda ratio of 0.06 (normal: 0.26-1.65 mg/dL). Cardiac magnetic resonance imaging (MRI) with gadolinium-based delayed contrast administration was significant for large areas of delayed enhancement involving both the left and right ventricular myocardia, indicating significant wall thickening (Figure 1). The left ventricle demonstrated a further depressed ejection fraction of 29% along with evidence of biatrial enlargement.

**Figure 2:**
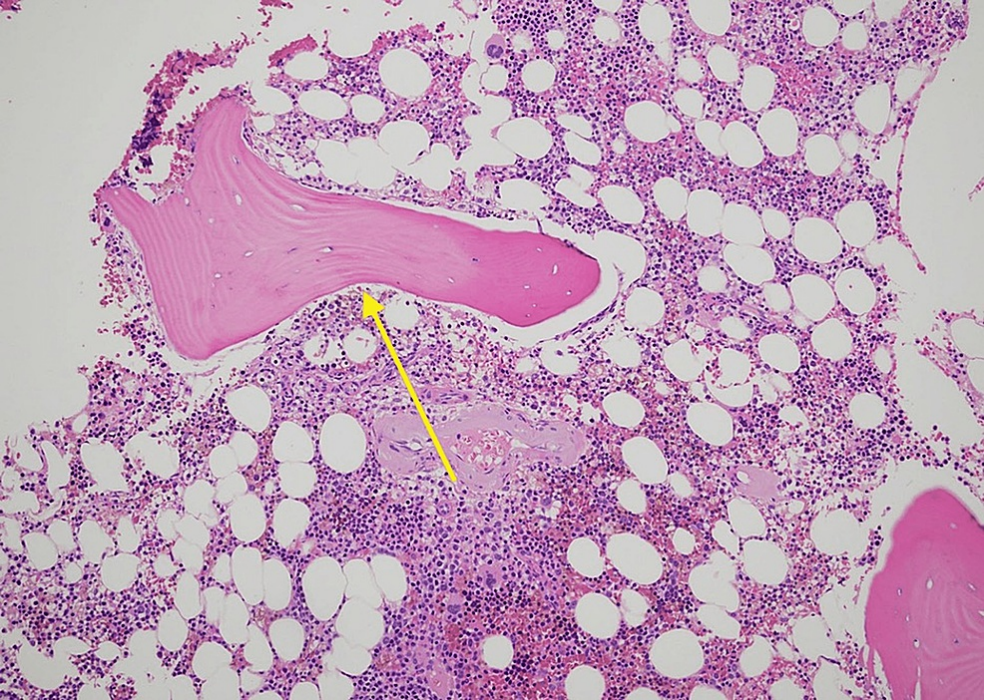
Hematoxylin and eosin (H&E) staining of the bone marrow biopsy shows a perivascular pink, waxy substance characteristic of amyloid

A bone marrow biopsy with hematoxylin and eosin (H&E) stain showed perivascular positivity with a thickened and waxy morphological appearance (Figure 2).

The diagnosis of amyloidosis was further supported by the subsequently positive Congo red stain with "apple-green birefringence" on a liver biopsy. She was undergoing a heart transplant evaluation when she was started on a combination treatment of cyclophosphamide, bortezomib, and dexamethasone. Despite treatment, the patient required additional pressor support and experienced more frequent episodes of non-sustained and sustained ventricular tachycardia. On day 14 of admission, she suffered a cardiac arrest unresponsive to cardiopulmonary resuscitation and was eventually pronounced deceased.

## Discussion

Among the subtypes of amyloidosis, light-chain (AL) and transthyretin (ATTR) amyloidosis are implicated in over 90% of cardiac cases [9], with AL patients typically exhibiting worse outcomes [10]. Cardiac involvement is said to be the most important determinant of poor prognosis in these patients [11]. Specifically, AL patients left untreated have a median survival of just six months [12], which may be partially explained starting at the cellular level. The pathophysiology of AL amyloidosis is thought to be due to chromosome instability leading to immunoglobulin chain translocation [13]. The most common translocation is suspected to cause plasma cell dyscrasia, which leads to the excessive production of misfolded light chains [2]. Progressive fibril deposition eventually contributes to end-organ damage in the heart, kidneys, and liver. About 50% of AL amyloid patients are said to suffer from heart failure due to diastolic dysfunction and restrictive cardiomyopathy [14, 10]. The amyloid protein consists of several glycoproteins, most notably serum amyloid glycoproteins, that provide its strength and resistance to degradation [15]. The insoluble array of fibril deposition is said to elevate the "defibrillation threshold" of myocytes, thereby increasing a patient’s susceptibility to life-threatening arrhythmias [16], as depicted in the above case. In this case, the patient’s fibrotic myocardium sustained pulseless ventricular tachycardia despite progressive augmentation of defibrillation voltage. Given the delayed presentation of this case, it appears that the disease was too advanced for treatment measures to exhibit even a considerable improvement in outcomes. The time between the first symptoms of heart failure and the diagnosis of amyloidosis is a significant determinant of the patient's prognosis. In fact, a study published in the European Journal of Haematology found that, compared to early diagnosis, a delay in diagnosis of greater than 13 months from the time of symptom onset has at least a three-fold elevated risk of death [17]. Hence, a timely diagnosis is imperative. A clinical picture suggesting an infiltrative process must be confirmed via biopsy, as laboratory evaluation may not always indicate a definitive diagnosis.

As seen in the above case, the patient’s serum protein electrophoresis was normal, which can reportedly occur in up to 25% of patients, likely secondary to light chain filtration by the kidneys [18]. However, these values must not be neglected; routine measurement of serum-free light chains is often used by providers in assessing response to treatment [2]. Treatment is initially focused on symptom management and preventing further damage caused by light chain deposition. Myocardial thickening often leads to heart failure with both systolic and diastolic dysfunction. Depending on the stage of heart failure and symptom burden, guideline-directed medical therapy may be recommended by a cardiac specialist. Specific hematological therapy often includes a combination of medications to treat the effects of this plasma cell dyscrasia. Our patient was started on CyBorD, or a combination of cyclophosphamide, bortezomib, and dexamethasone, to mitigate the burden of light chain deposition. Some patients may be candidates for autologous stem cell transplantation; however, like our patient, patients with an ejection fracture under 40% may not be suitable candidates due to an elevated risk of decompensation [19].

Cardiac transplantation is generally the last step in the treatment of severe cardiac amyloidosis in patients who qualify. Cardiac transplantation is estimated to have a five-year survival rate of 75%, so a patient with a lower projected survival would not likely be a candidate. In these cases, cardiology and hematology specialists carefully collaborate on the decision to transplant, which may even be postponed until better optimization of the light chain burden with medical management [19].

## Conclusions

Cardiac AL amyloidosis is associated with an elevated mortality rate due to myocardial fibrosis. Increased fibrosis is associated with cardiogenic decline and a predisposition to ventricular arrhythmias. Though our patient’s delayed diagnosis was partly due to uncontrollable socioeconomic factors, her clinical course helps to demonstrate the risks associated with late recognition and treatment. Treatment options include guideline-directed medical therapy for heart failure, plasma-cell-directed therapies, stem cell transplants, and heart transplants. Therapeutic efficacy is directly correlated with the timing of the diagnosis. With this case, we hope to illustrate the rapidly progressive nature of cardiac amyloidosis and help promote earlier diagnosis.
